# Melatonin Ameliorates Valproic Acid-Induced Neurogenesis Impairment: The Role of Oxidative Stress in Adult Rats

**DOI:** 10.1155/2021/9997582

**Published:** 2021-11-12

**Authors:** Anusara Aranarochana, Apiwat Sirichoat, Wanassanun Pannangrong, Peter Wigmore, Jariya Umka Welbat

**Affiliations:** ^1^Department of Anatomy, Faculty of Medicine, Khon Kaen University, Khon Kaen, Thailand; ^2^Neurogenesis Research Group, Department of Anatomy, Faculty of Medicine, Khon Kaen University, Khon Kaen 40002, Thailand; ^3^School of Life Sciences, Medical School, Queen's Medical Centre, Nottingham University, Nottingham, UK

## Abstract

**Background:**

Valproic acid (anticonvulsant medication) has been found to inhibit histone deacetylase activity and suppress hippocampal neurogenesis, which causes memory impairment in both humans and rodents. The neurohormone melatonin, which regulates mammalian seasonal and circadian physiology, has recently been shown to have neuroprotective properties, counteracting memory impairment associated with VPA-caused hippocampal neurogenesis reduction. This study is aimed at investigating the molecular mechanisms of melatonin associated with VPA-induced hippocampal neurogenesis and memory impairment.

**Methods:**

Male Spraque-Dawley rats received VPA (300 mg/kg) twice daily or melatonin (8 mg/kg/day) or some rats were given melatonin for 14 days during VPA administration.

**Results:**

The VPA-treated rats showed a significant increase in malondialdehyde (MDA) levels in the hippocampus and p21-positive cells in the subgranular zone (SGZ) of the dentate gyrus (DG) but decreased superoxide dismutase (SOD), catalase, and glutathione peroxidase (GPx) activities. Moreover, VPA significantly decreased levels of nestin, Notchl, nuclear factor erythroid 2-related factor 2 (Nrf2), doublecortin (DCX), sex determining region Y-box 2 (SOX2), and brain-derived neurotrophic factor (BDNF).

**Conclusions:**

We found that melatonin was able to counteract these neurotoxic effects, acting as a neuroprotectant in VPA-induced memory hippocampal neurogenesis impairment by preventing intracellular oxidative stress and increasing antioxidant activity.

## 1. Background

The brain tissue is particularly vulnerable to oxidative stress, which causes high brain oxygen consumption, resulting in increased reactive oxygen species (ROS) generation including byproducts of lipid peroxidation, which can impair brain function [1]. ROS produces oxidative stress in the brain, which injures glial cells and neurons, leading to neuronal damage [2]. Oxidative stress is a result of an imbalance between ROS and antioxidant systems, which is an important cause of neuronal cell death and memory loss in neurodegenerative disorders [2, 3]. The hippocampal DG, which is responsible for learning and memory performance, is a region of the brain that is especially sensitive to ROS-induced oxidative stress [4]. Previous research has demonstrated that functional interactions between the hippocampus and prefrontal cortex play a role in cognitive performance, especially with regard to spatial working memory [5]. The oxidative damage underlying neuronal cell death, which results from increased oxidative stress and decreases in antioxidative agents in the hippocampus and prefrontal cortex, contributes to memory impairment [6, 7]. In animal models, exposure to valproic acid (VPA), an anticonvulsant drug, causes memory impairment associated with aberrations in hippocampal neurogenesis, specifically lower hippocampal cell proliferation, cell survival, and immature neuron numbers [8]. The adverse effects of VPA treatment in both patients [9] and animal models [10] are associated with oxidative stress and reductions in antioxidant enzymes including glutathione peroxidase (GPx), superoxide dismutase (SOD), and catalase [11]. VPA is a class I selective histone deacetylase inhibitor, which suppresses gene expression [12], and VPA-induced hyperacetylation of DNA leads to suppression of cell proliferation [13]. Additionally, VPA causes cell growth arrest through the upregulating of the cyclin-dependent kinase inhibitor, p21Cip1/WAF1 (p21) [14] and concurrent increases in intracellular apoptosis [15]. Previous reports have shown melatonin and its metabolites to be potent antioxidant agents due to their functioning as direct free radical scavengers [16, 17]. Melatonin also ameliorates free radical-induced damage caused by oxidative stress from diverse agents such as neural toxins, metals, and irradiation [18] and can reduce hippocampal cellular death through the direct scavenging ROS [19]. Melatonin treatment also leads to stimulation of neural stem cell (NSC) proliferation and differentiation [20]. Both in vitro and in vivo studies have shown that exogenous melatonin can accelerate cell proliferation, improve newborn cell survival, and increase the number of immature neurons during hippocampal neurogenesis [21]. A recent study we conducted confirmed the neuroprotective effects of melatonin in counteracting the memory impairment associated with VPA-induced hippocampal neurogenesis reduction [8]. This study was designed to determine whether there are other mechanisms by which melatonin may enhance the ability of antioxidant enzymes to neutralize the adverse effects of VPA. We assessed hippocampal neurogenesis-related protein expression to demonstrate the neuroprotective effects of melatonin on VPA-induced hippocampal neurogenesis impairment.

## 2. Methods

### 2.1. Animals and Drug Administration

Male Sprague Dawley rats (age: 4-5 weeks, weight: 180-220 g; obtained from Nomura Siam International Co., Ltd., Bangkok, Thailand) were randomly allocated to four groups: control, VPA, melatonin, and preventive. The rats were habituated under a 12 h (7:00 AM to 7:00 PM) light-dark cycle with food and water for one week prior to the experiments. All experimental procedures were approved by the Khon Kean University Ethics Committee in Animal Research (project number: IACUC-KKU 19/61).

**Figure 1:**
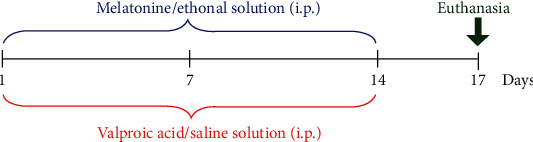
Timeline of drug administration. The red bracket represents the time for intraperitoneal injection of VPA/saline solution. The blue bracket represents the period for intraperitoneal injection of melatonin/ethanol solution. Animals were euthanized, and the brains were collected on day 17 as shown by the green arrow.

VPA at a dose of 300 mg/kg was dissolved in 0.9% saline and administered as two daily intraperitoneal injections for 14 days [8, 22]. Melatonin at a dose of 8 mg/kg was dissolved in ethanol and then diluted in 0.9% saline to obtain the final working concentration and administered daily by intraperitoneal injection for 14 days (Figure 1). Rats were each randomly assigned one of four groups (12 rats per group) as follows:

- Control group: received 0.9% saline at 10 a.m. and 3 p.m. and ethanol at 7 p.m.
- VPA group: received VPA at 10 a.m. and 3 p.m.
- Melatonin group: received melatonin at 7 p.m.
- Preventive group: received VPA at 10 a.m. and 3 p.m. and melatonin at 7 p.m.

### 2.2. Tissue Preparation

Rats were euthanized 3 days after discontinuation of drug administration, and their brains were embedded in optimal cutting temperature (OCT) compound and then snap-frozen. The frozen brains were kept in a freezer at -80°C for immunofluorescent study. The hippocampus from the other half of the brain was rapidly snap-frozen using liquid nitrogen and stored at -80°C for Western immunoblotting and antioxidant assay.

### 2.3. Immunofluorescent Staining of p21

Frozen hippocampi were serially cut (40 *μ*m) on the coronal plane using a freezing microtome. The sections were incubated with mouse monoclonal anti-p21 antibody overnight at 4°C. They were then incubated with Alexa fluor 488-conjugated rabbit anti-mouse IgG and counterstained with PI for 30 sec. All sections were quantified at 40x under a Nikon ECLIPSE 80i fluorescence microscope running NIS-Element AR 3.2 software. Following a systematic random sampling method [23], nine hippocampal sections were selected from every eighth section along the entire length of the DG, and the number of p21 immunopositive cells in each was calculated as raw data and then multiplied by 8 to estimate the total number [8, 24].

### 2.4. Hippocampal Protein Expression Study

Hippocampal tissue samples were homogenized using lysis buffer for Western blotting as previously described [24]. Fifty micrograms of protein concentration per lane were loaded for electrophoreses on 10% SDS-polyacrylamide gels for nestin, Notch1, and nuclear factor erythroid 2-related factor 2 (Nrf2) expression appraisal, while 12% SDS-polyacrylamide gels were used to assess doublecortin (DCX), sex determining region Y-box 2 (SOX2), and brain-derived neurotrophic factor (BDNF) expression. The separated proteins were transferred from the gel onto suitable nitrocellulose blotting membranes. Immunoblots were probed overnight at 4°C using the following primary antibodies: anti-nestin, anti-Notch1, anti-Nrf2, anti-DCX, anti-SOX2, anti-BDNF, and monoclonal mouse anti-GAPDH as an internal loading control. The blots were then incubated with horseradish peroxidase-conjugated secondary antibodies (goat anti-mouse, goat anti-rabbit, and rabbit anti-goat), and the signals from the protein blots were analyzed using an ECL detection system. The density of each protein band was quantified using ImageJ software. Optical density expressions of all data were exhibited as a ratio of GAPDH expression.

### 2.5. Determination of Antioxidant Activity and Lipid Peroxidation Markers

Hippocampal tissue samples were homogenized using deionized water in an ice bath for 30 min and then centrifuged to separate and allow for collection of the supernatants. The SOD, GPx, catalase, and MDA levels were investigated using a chemical colorimetric method. MDA levels were determined by thiobarbituric acid reaction substances (TBARS) analysis. In acidic conditions, MDA can react with thiobarbituric acid (TBA) at 95°C, thus contributing to nucleophilic addition reaction to generate a red color, which was detected at 532 nm [25]. MDA level was expressed as nmol per milligram of protein using 1,1,3,3-tetraethoxypropane as a standard solution. Catalase enzyme activity was assessed by measuring the decomposition of hydrogen peroxide into oxygen and water. The hydrogen peroxide decomposition was catalyzed using catalase. In order to determine the effectiveness with regard to decomposition of the catalase in the supernatant, potassium permanganate was added to react with the residuals of the hydrogen peroxide, which was measured at OD 540 nm [26]. GPx activity was measured by coupled reaction catalyzed by glutathione reductase (GR). GPx catalyzes the reduction of H_2_O_2_ by glutathione (GSH) into H_2_O and simultaneously reduces GSH to GSSG. GSSG can react with 5,5′-dithiobis-(2-nitrobenzoic acid) (DTNB) to form yellow-colored chromophores, which are detected at 405 nm. The generated GSSG is reduced to GSH, using nicotinamide-adenine dinucleotide phosphate (NADPH) by GR. The GPx activity is proportional to the decrease of NADPH [27]. SOD activity was evaluated based on the capacity of SOD to inhibit the autoxidation-induced by the xanthine-xanthine oxidase reaction. Xanthine oxidase produces superoxide radicals, which reduce cytochrome c to its oxidized form. This reduction was detected at 550 nm. One unit of SOD can inhibit the rate of cytochrome c reduction by 50% to compete with the superoxide radicals [28]. The activities of SOD, CAT, and GPx were calculated from linear equations, which were referenced to the activity of each enzyme and reported as units of enzymatic activity per milligram of protein.

### 2.6. Statistical Analysis

All statistical parameters were tested using GraphPad Prism and expressed as mean ± standard error of mean (SEM). The one-way analysis of variance (ANOVA) was used to analyze data, and post hoc comparisons between groups were subsequently made using a Bonferroni test. A *P* value of <0.05 was considered statistically significant.

## 3. Results

### 3.1. Effects of VPA and Melatonin on the p21-Positive Cell Count in the Hippocampus

**Figure 2:**
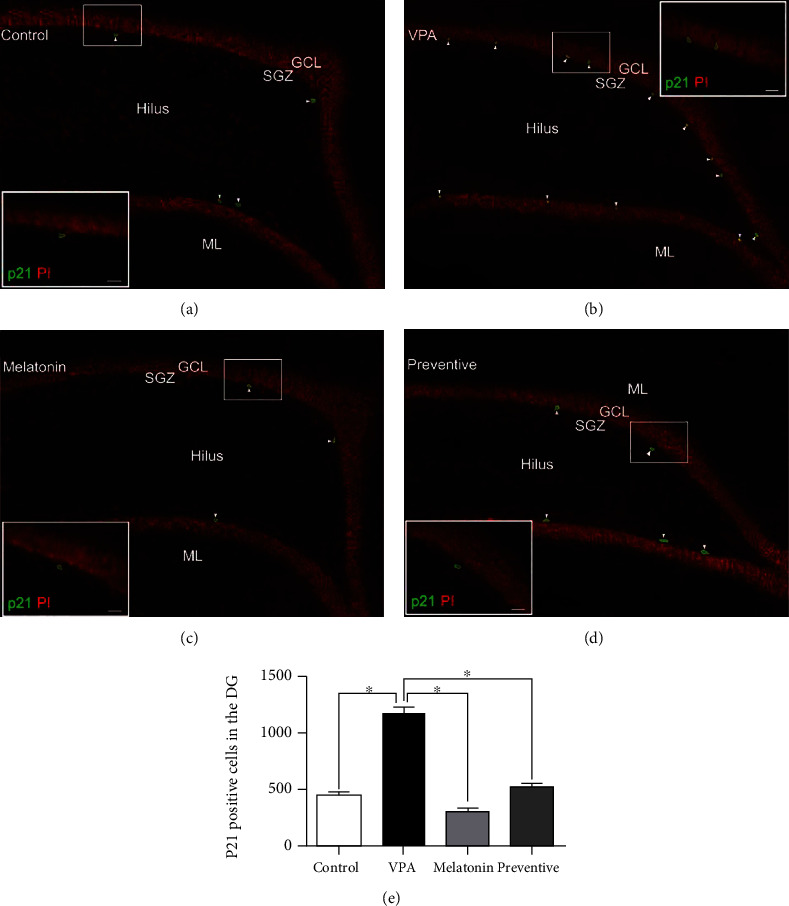
Images of p21-positive cells (green) in the SGZ of the hippocampal DG by group (a–d). All nuclei were counterstained with propidium iodide (red). Arrowheads indicate p21-positive cells in the DG (scale bars: 100 *μ*m). The inserted figures show p21 immunostaining at high magnification (scale bars: 50 *μ*m). The number of p21-positive cells was significantly lower in the VPA group than in the control, melatonin, and preventive groups (^∗^*p* < 0.05, (e)).

Immunofluorescent staining of p21 was performed to investigate cell cycle arrest related to cell damage (Figure 2). When compared with controls, p21-positive cell counts were significantly higher in VPA-treated animals and significantly lower in those given melatonin alone (*p* < 0.05). In addition, the number of p21-positive cells in the preventive group was significantly lower than that in the VPA group (*p* < 0.05). This suggests that melatonin may prevent cell damage caused by VPA.

### 3.2. Effects of VPA and Melatonin on the Expression of Nestin, Notchl, Nrf2, DCX, SOX2, and BDNF

**Figure 3:**
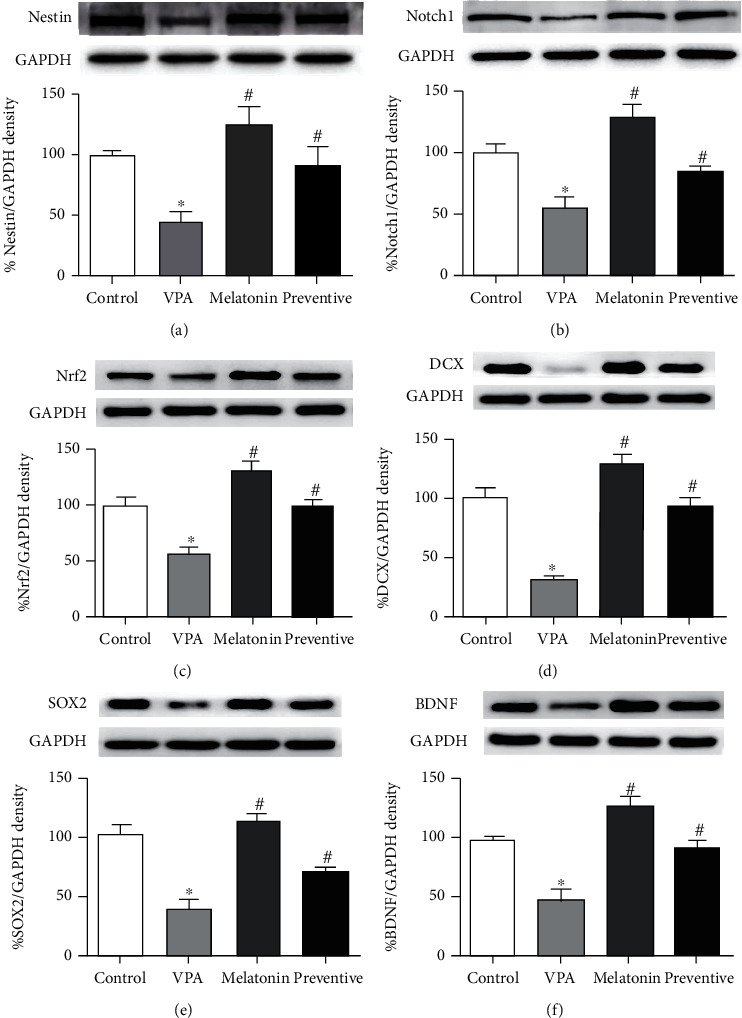
The expression of nestin (a), Notch1 (b), Nrf2 (c), DCX (d), SOX2 (e), and brain-derived neurotrophic factor or BDNF (f) in the hippocampus were determined by Western blot analysis (mean ± SE). ^∗^*p* < 0.05 compared to the control group, ^#^*p* < 0.05 compared to the VPA-treated group.

Expression levels of nestin, Notchl, Nrf2, DCX, SOX2, and BDNF in the hippocampus were determined by Western blot analysis. Nestin expression was significantly lower in the VPA-treated group than in the control (*p* = 0.0007, Figure 3(a)) and preventative groups (*p* < 0.05). This shows that coadministration with melatonin protected against decreases in nestin protein levels caused by VPA. Animals that received VPA also showed a significant decrease in Notch1 expression compared to the control animals (*p* < 0.0001, one-way ANOVA, Figure 3(b)) and those in the preventative group (*p* < 0.05). This suggests that melatonin coadministration can ameliorate VPA-induced reductions in Notch1 expression in the hippocampus. In addition, Nrf2 levels in the VPA-treated group were significantly lower than those in both the control (*p* < 0.0001, one-way ANOVA, Figure 3(c)) and preventive groups (*p* < 0.05). These results imply that coadministration with melatonin is able to upregulate Nrf2 levels associated with the intracellular antioxidant system, preventing the adverse effects of VPA. DCX expression in VPA-treated animals was significantly lower than that in the control group (*p* < 0.0001, one-way ANOVA, Figure 3(d)) and the preventive group (*p* < 0.05). This suggests that VPA caused reductions in DCX levels in the hippocampus, which were counteracted by melatonin coadministration.

Animals in the VPA-treated group had significantly lower levels of SOX2 compared to those in both the control group (*p* < 0.0001, one-way ANOVA, Figure 3(e)) and the preventive group (*p* < 0.05), indicating that coadministration with melatonin prevented VPA-induced decreases in SOX2 protein. Animals that underwent VPA treatment also had significantly lower BDNF levels than those in the control (*p* < 0.0001, Figure 3(f)) and preventive groups. These results demonstrate that melatonin coadministration can protect against BDNF protein deficiency caused by VPA.

### 3.3. Effects of VPA and Melatonin on MDA Levels and SOD, Catalase, and GPx Activities

**Figure 4:**
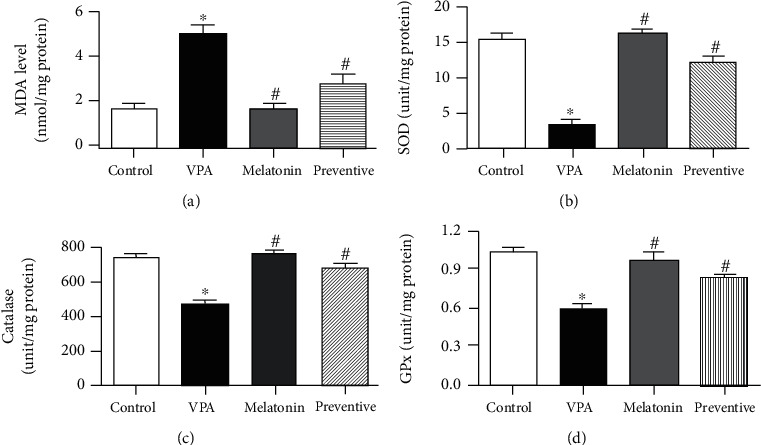
Malondialdehyde (MDA) levels and superoxide dismutases (SOD), catalase, and glutathione peroxidase (GPx) activities (mean ± SEM) in hippocampal tissue. ^∗^*p* < 0.05 compared to the control group, ^#^*p* < 0.05 compared to the VPA-treated group.

**Figure 5:**
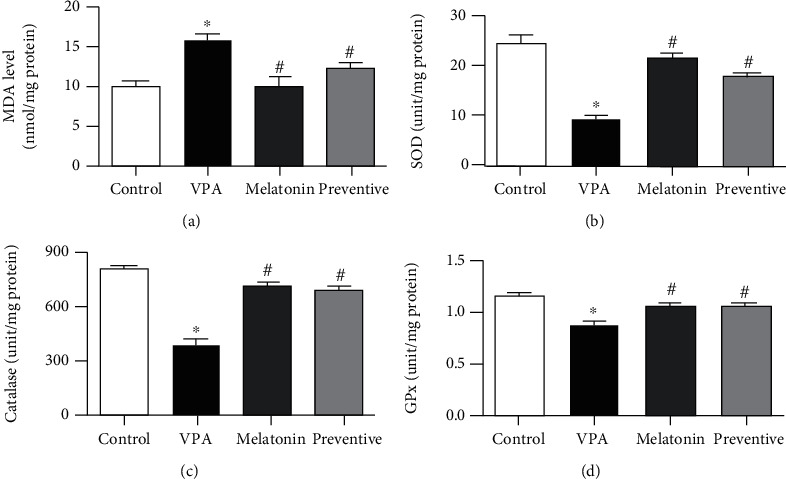
The level of malondialdehyde (MDA) and the activity of superoxide dismutase (SOD), catalase, and glutathione peroxidase (GPx) (mean ± SEM) in the prefrontal cortex. ^∗^*p* < 0.05 compared to the control group, ^#^*p* < 0.05 compared to the VPA-treated group.

MDA levels in the VPA-treated group were significantly higher than those in the control (*p* < 0.0001, Figure 4(a)), melatonin, and preventive groups (*p* < 0.05). MDA levels in the latter three groups did not differ significantly. This suggests that melatonin coadministration can neutralize the impact of VPA on hippocampal MDA levels. SOD activity in the hippocampus in the VPA-treated group was significantly lower than in the control (*p* < 0.0001, Figure 4(b)) and preventive groups (*p* < 0.05), indicating that melatonin coadministration can ameliorate the impact of VPA on SOD activity in the hippocampus. Likewise, the VPA-treated animals showed significant decreases in catalase activity when compared to the control (*p* < 0.0001, Figure 4(c)), melatonin, and preventive groups (*p* < 0.05), suggesting that melatonin coadministration can protect against the adverse effects of VPA on hippocampal catalase activity. In addition, GPx activity was significantly lower in the VPA-treated group compared with that in the control (*p* < 0.0001, Figure 4(d)) and preventive groups (*p* < 0.05). This demonstrates melatonin's ability to protect against the declines in hippocampal GPx activity caused by VPA. Prefrontal cortex MDA levels were significantly higher in the VPA-treated animals (*p* < 0.0001, Figure 5(a)) but normal in the preventive group (Figure 5(a)). VPA-treated animals also exhibited significant reductions in SOD (*p* < 0.0001, Figure 5(b)), catalase (*p* < 0.0001, Figure 5(c)), and GPx (*p* < 0.0001, Figure 5(d)) activity in the prefrontal cortex, all of which were higher in the preventive group (Figures 5(b)–5(d)). This indicates that melatonin was able to prevent oxidative stress and defend against the negative effects of VPA in the prefrontal cortex.

## 4. Discussion

Valproic acid is clinically used as a conventional antiepileptic drug to treat patients with seizures. VPA treated alone inhibits different types of seizures by reducing the severity of convulsions resulting in the suppression of behavioural changes caused by seizures [29]. Another finding has shown that VPA (nonprotective dose) potentially exhibits anticonvulsive activities in a model of metaphit-induced seizures with combination by delta-sleep-inducing peptide (somnogenic nonapeptide) [30]. Although VPA has low toxicity and a good safety profile, it has been reported a variety of serious effects like impairments of memory. Either an overabundance of ROS or antioxidant deficiencies can lead to oxidative stress, which is significantly associated with neurodegeneration including cognitive impairment [31]. Transcription factor Nrf2 plays an important role in promoting key antioxidant enzymes and thus reducing ROS and protecting against ROS-induced cell damage. For this reason, Nrf2 has been identified as a therapeutic target in neurodegenerative diseases [32, 33]. Nrf2 also regulates hippocampal neurogenesis by promoting the proliferation and survival of NSCs and enhancing neuronal differentiation of neural progenitor cells (NPCs) [34]. Previous research has shown that Nrf2 improves learning by protecting hippocampal neurons against amyloid-beta toxicity [35]. Our study found that treatment with VPA decreased Nrf2 expression, which is consistent with the results of a previous study [36]. A growing number of reports have described the protective effects of melatonin against intracellular oxidative damage through increased Nrf2 expression [37–39]. Likewise, this study demonstrated that coadministration with melatonin significantly increased Nrf2 levels in the hippocampus under VPA-induced oxidative stress.

Clinical dosages of VPA also induce apoptotic neurodegeneration in various areas of the brain including the hippocampus and prefrontal cortex [40]. Side effects during VPA treatment are associated with oxidative stress and reductions in antioxidant enzymes including glutathione peroxidase, superoxide dismutase, and catalase [10, 11]. Our study supports these associations, in that VPA-caused oxidative stress resulted in increases in both prefrontal and hippocampal MDA levels, the end products of lipid peroxidation. Furthermore, treatment with VPA decreased SOD, GPx, and catalase activities, demonstrating that it causes intracellular oxidative stress and abatement of antioxidant enzymes, both of which are correlated with memory impairment. Melatonin and its metabolites link to the melatonin antioxidant pathway in the rat brain and act as reducing agents, which neutralize free radical activities through electron donation to radicals, a process that results in protection against oxidative damage to the neuronal cells [41, 42]. In this study, melatonin coadministration significantly increased SOD, GPx, and catalase activities and lowered MDA levels. These results are consistent with those of previous studies that melatonin relieves the effects of oxidative stress and neutralizes the decreases in antioxidant enzyme, SOD, GPx, and catalase activities induced by oxidative damage [43, 44], which suggests that it augments the defensive system and attenuates lipid peroxidation. It is possible that melatonin exhibits its neuroprotective effects by facilitating the antioxidant system and inhibiting intracellular oxidative stress, which prevents memory impairment caused by VPA.

The cellular response mechanism to DNA-oxidative damaging agents relies on the expression of p21 to induce cell cycle arrest [45]. We found that oxidative stress triggered by VPA leads to stimulation of p21 expression, resulting in the suppression of hippocampal neurogenesis. Previous studies have suggested that VPA treatment interferes in cell cycle arrest by increasing p21 expression levels [46]. The presence of p21 blocks the proliferation of hippocampal granule neurons and is consequently responsible for restriction of cell cycle progression in the hippocampal SGZ [47]. There is an evidence that melatonin prevents the upregulation of p21 caused by irradiation-induced oxidative damage [48], which is in line with our findings that melatonin coadministration protects against VPA-induced hippocampal neurogenesis impairment by reducing p21 levels.

In the context of neuronal migration and differentiation in the DG, DCX-expressing immature neurons are essential in learning and memory formation [49]. In the present study, animals receiving VPA alone had significantly lower DCX protein levels than the controls. This is similar to the results of our previous research, which found that memory impairment caused by VPA in an animal model is related to decreases in the population of DCX-positive cells in the hippocampal DG [50]. This study also showed that animals received melatonin coadministration had significantly higher DCX protein levels than those treated with VPA alone, which is also in line with our previous study. In addition, melatonin prevented reductions of cell proliferation and damage to immature neurons caused by dexamethasone [51]. This indicates that melatonin modulates adult hippocampal neurogenesis by affecting the proportion of newly developed immature neurons, which counteracts the cognitive effects of VPA and prevents memory impairment.

Adult hippocampal neurogenesis is regulated by BDNF, which influences the proliferation, survival, and morphology of granule cells in the SGZ of the DG; particularly, BDNF augments hippocampal neurogenesis, resulting in improved cognitive performance in animal models [52]. In this study, VPA treatment alone caused a significant reduction in BDNF levels, a result that is consistent with the findings of our previous studies [22, 50] that VPA-induced downregulation of BDNF in the hippocampus is related to impairment of hippocampal neurogenesis and memory. A recent study has shown that melatonin prevents hippocampal damage and cognitive dysfunction against BCCAO-induced vascular dementia by altering BDNF expression [53]. Our study showed that BDNF levels in animals receiving melatonin coadministration were significantly higher than in VPA-treated animals. This suggests that melatonin might prevent VPA-induced decreases in cell proliferation, cell survival, and memory by upregulating BDNF levels in the hippocampus.

Notch1 is a large transmembrane protein receptor, which regulates and maintains NSC properties [54]. Hippocampal NSCs produce high levels of Notch1, which is associated with adult neurogenesis in the SGZ of the hippocampus [55]. Decreases in Notch1 expression are associated with the impairment of neurogenesis in the hippocampal DG and of spatial working memory [22]. This study found that VPA treatment decreased levels of Notchl expression, a finding that is consistent with those of our previous reports that VPA reduced Notch1 expression, which was in turn associated with decreases in cell proliferation and spatial memory [22, 56]. However, we found that the decreases in Notchl caused by VPA can be prevented by coadministration with melatonin. This result supports previous findings that melatonin ameliorates synapse dysfunction and spatial memory impairment by increasing Notch1 expression in the hippocampus [57]. This suggests that melatonin might play an important role in the prevention of memory and hippocampal neurogenesis impairment through the upregulation of Notchl expression in the adult hippocampus. Additionally, the enhancement of signaling from the Notch1 pathway activates SOX2 promoters, which promote the proliferation and differentiation of adult hippocampal NSCs [58]. SOX2 is a transcription factor and preserves NSC characteristics including neural stem cell proliferation, survival, and differentiation [59]. The expression of SOX2 is downregulated by VPA [60], which is similar to the reductions in hippocampal SOX2 levels induced by the antiproliferative effect of VPA in the present study. Melatonin also increases SOX2 levels, which leads to the promotion of cell proliferation and differentiation [61] and enhances the expression of SOX2 and Nrf2. In this study, coadministration with melatonin resulted in significantly higher SOX2 expression when compared to VPA treatment alone. These findings suggest that melatonin has the ability to upregulate SOX2 expression in hippocampal NSCs, which appears to counteract memory and hippocampal neurogenesis impairment.

Nestin protein has commonly been used as a marker for NSCs and NPCs in the adult brain [62]. A recent report suggests that NSCs require nestin for their self-renewal activity and survival [63]. Our study found that treatment with VPA alone decreased nestin protein expression. This is consistent with the results of our previous study, which found that the restriction of nestin expression caused by treatment with the chemotherapeutic drug, 5-fluorouracil, was related to decreases in NPCs in adult rats [24]. However, nestin expression was significantly higher in animals that underwent melatonin coadministration than in those treated with VPA alone. This suggests that melatonin might prevent reductions in NPCs by promoting the expression of nestin protein. The protective effect of melatonin found in this study is consistent with the findings of previous studies that melatonin upregulates the expression of nestin, which is associated with the proliferation and survival of stem cells and NSC differentiation [61, 64]. Moreover, the expression of nestin is controlled by the expression of SOX2, which is necessary for the maintenance of NSCs and subsequent neurogenesis and is concomitant with the expression of nestin in NSCs [65].

## 5. Conclusions

The results of this study confirm the neuroprotective effects of melatonin in the SGZ of the hippocampal DG by enhancing the functions of the antioxidant enzymes and prohibiting lipid peroxidation. We also found that coadministration with melatonin is able to counteract memory impairment associated with VPA-induced increases in p21 and reductions in hippocampal neurogenesis. Melatonin coadministration also prevents the side effects of VPA on hippocampal neurogenesis by increasing BNDF, Notchl, SOX2, nestin, DCX, and Nrf2 expression, which leads to improvements in memory. All of this suggests that melatonin may be effective in preventing memory impairment induced by VPA.

## Data Availability

The data used to support the finding of this study are included within the article.

## Abbreviations

VPA: Valproic acid
MDA: Malondialdehyde
SGZ: Subgranular zone
DG: Dentate gyrus
SOD: Superoxide dismutase
GPx: Glutathione peroxidase
Nrf2: Nuclear factor erythroid 2-related factor 2
DCX: Doublecortin
SOX2: Sex determining region Y-box 2
BDNF: Brain-derived neurotrophic factor
ROS: Reactive oxygen species
p21: p21Cip1/WAF1
OCT: Optimal cutting temperature
PI: Propidium iodide
SDS: Sodium dodecyl sulfate
GAPDH: Glyceraldehyde-3-phosphate dehydrogenase
ECL: Enhanced chemiluminescence
TBARS: Thiobarbituric acid reaction substances
TBA: Thiobarbituric acid
OD: Optical density
GR: Glutathione reductase
GSH: Glutathione
GSSG: Glutathione disulfide
DTNB: 5,5′-Dithiobis-(2-nitrobenzoic acid)
NADPH: Nicotinamide-adenine dinucleotide phosphate
SEM: Standard error of mean
ANOVA: Analysis of variance
NSC: Neural stem cell
NPC: Neural progenitor cell
DNA: Deoxyribonucleic acid
BCCAO: Bilateral common carotid artery occlusion.
